# Supplementary material for: Modeling glioblastoma heterogeneity as a dynamic network of cell states
Source: Mol Syst Biol. 2021 Sep 16;17(9):e10105. doi: 10.15252/msb.202010105 (PMC8444284; doi:10.15252/msb.202010105)
Supplement: Supplementary file 6 — Source Data for Figure 5 [file MSB-17-e10105-s004.zip › Figure5A_sourcedata/GSEA_3017/nosigdb_stateB.GseaPreranked.1622795021725/DOROSZKO_U3180MG_CLUSTER_3.html]

Details for gene set DOROSZKO\_U3180MG\_CLUSTER\_3[GSEA]

|  || Dataset | state43017 |
| Phenotype | NoPhenotypeAvailable |
| Upregulated in class | na\_pos |
| GeneSet | DOROSZKO\_U3180MG\_CLUSTER\_3 |
| Enrichment Score (ES) | 0.5211078 |
| Normalized Enrichment Score (NES) | 3.3311236 |
| Nominal p-value | 0.0 |
| FDR q-value | 0.0 |
| FWER p-Value | 0.0 |
Table: GSEA Results Summary

  

Fig 1: Enrichment plot: DOROSZKO\_U3180MG\_CLUSTER\_3      
 Profile of the Running ES Score & Positions of GeneSet Members on the Rank Ordered List

  

| PROBE | GENE SYMBOL | GENE\_TITLE | RANK IN GENE LIST | RANK METRIC SCORE | RUNNING ES | CORE ENRICHMENT || 1 | HIST1H4C |  |  | 0 | 1.373 | 0.0121 | Yes |
| 2 | HIST1H1D |  |  | 2 | 1.180 | 0.0206 | Yes |
| 3 | TOP2A |  |  | 3 | 1.079 | 0.0301 | Yes |
| 4 | HIST1H1E |  |  | 5 | 0.983 | 0.0369 | Yes |
| 5 | RRM2 |  |  | 7 | 0.875 | 0.0426 | Yes |
| 6 | FAM111A |  |  | 10 | 0.813 | 0.0459 | Yes |
| 7 | UBE2C |  |  | 11 | 0.802 | 0.0530 | Yes |
| 8 | TPX2 |  |  | 12 | 0.801 | 0.0601 | Yes |
| 9 | NUSAP1 |  |  | 13 | 0.801 | 0.0672 | Yes |
| 10 | HMGB2 |  |  | 14 | 0.786 | 0.0741 | Yes |
| 11 | FBXO5 |  |  | 15 | 0.785 | 0.0810 | Yes |
| 12 | NCAPG |  |  | 16 | 0.778 | 0.0879 | Yes |
| 13 | SPC25 |  |  | 17 | 0.763 | 0.0946 | Yes |
| 14 | PRC1 |  |  | 18 | 0.756 | 0.1013 | Yes |
| 15 | CDCA2 |  |  | 19 | 0.751 | 0.1080 | Yes |
| 16 | ESCO2 |  |  | 20 | 0.742 | 0.1145 | Yes |
| 17 | TACC3 |  |  | 21 | 0.741 | 0.1211 | Yes |
| 18 | GTSE1 |  |  | 22 | 0.732 | 0.1275 | Yes |
| 19 | HJURP |  |  | 23 | 0.731 | 0.1340 | Yes |
| 20 | KIF23 |  |  | 24 | 0.721 | 0.1404 | Yes |
| 21 | ASPM |  |  | 26 | 0.695 | 0.1445 | Yes |
| 22 | NDC80 |  |  | 27 | 0.695 | 0.1507 | Yes |
| 23 | ATAD2 |  |  | 28 | 0.685 | 0.1567 | Yes |
| 24 | KIF15 |  |  | 29 | 0.684 | 0.1628 | Yes |
| 25 | ARHGAP11A |  |  | 30 | 0.683 | 0.1688 | Yes |
| 26 | PCLAF |  |  | 31 | 0.681 | 0.1748 | Yes |
| 27 | SGO1 |  |  | 32 | 0.677 | 0.1808 | Yes |
| 28 | KPNA2 |  |  | 33 | 0.676 | 0.1868 | Yes |
| 29 | CENPF |  |  | 34 | 0.670 | 0.1927 | Yes |
| 30 | KNL1 |  |  | 35 | 0.666 | 0.1986 | Yes |
| 31 | SGO2 |  |  | 36 | 0.664 | 0.2045 | Yes |
| 32 | KIF4A |  |  | 37 | 0.662 | 0.2103 | Yes |
| 33 | RAD51AP1 |  |  | 40 | 0.657 | 0.2122 | Yes |
| 34 | SMC4 |  |  | 41 | 0.655 | 0.2180 | Yes |
| 35 | MELK |  |  | 42 | 0.655 | 0.2238 | Yes |
| 36 | NUF2 |  |  | 44 | 0.651 | 0.2276 | Yes |
| 37 | KIFC1 |  |  | 47 | 0.645 | 0.2293 | Yes |
| 38 | DHFR |  |  | 49 | 0.638 | 0.2330 | Yes |
| 39 | NCAPG2 |  |  | 50 | 0.636 | 0.2386 | Yes |
| 40 | CDCA5 |  |  | 52 | 0.632 | 0.2423 | Yes |
| 41 | CKAP2 |  |  | 53 | 0.628 | 0.2478 | Yes |
| 42 | CCNA2 |  |  | 54 | 0.626 | 0.2533 | Yes |
| 43 | FANCI |  |  | 56 | 0.615 | 0.2568 | Yes |
| 44 | MYBL2 |  |  | 58 | 0.612 | 0.2603 | Yes |
| 45 | CKAP2L |  |  | 59 | 0.611 | 0.2657 | Yes |
| 46 | CENPU |  |  | 60 | 0.605 | 0.2710 | Yes |
| 47 | CENPK |  |  | 62 | 0.603 | 0.2744 | Yes |
| 48 | TYMS |  |  | 63 | 0.601 | 0.2797 | Yes |
| 49 | BUB1B |  |  | 64 | 0.596 | 0.2850 | Yes |
| 50 | CCDC34 |  |  | 65 | 0.595 | 0.2902 | Yes |
| 51 | KIF2C |  |  | 66 | 0.591 | 0.2954 | Yes |
| 52 | SMC2 |  |  | 68 | 0.586 | 0.2987 | Yes |
| 53 | CDK1 |  |  | 69 | 0.585 | 0.3038 | Yes |
| 54 | H2AFX |  |  | 71 | 0.576 | 0.3070 | Yes |
| 55 | CKS1B |  |  | 72 | 0.575 | 0.3120 | Yes |
| 56 | CIP2A |  |  | 73 | 0.574 | 0.3171 | Yes |
| 57 | RACGAP1 |  |  | 75 | 0.572 | 0.3202 | Yes |
| 58 | FOXM1 |  |  | 76 | 0.570 | 0.3252 | Yes |
| 59 | MAD2L1 |  |  | 77 | 0.569 | 0.3303 | Yes |
| 60 | DBF4 |  |  | 79 | 0.567 | 0.3333 | Yes |
| 61 | CKS2 |  |  | 83 | 0.558 | 0.3324 | Yes |
| 62 | AURKB |  |  | 89 | 0.548 | 0.3274 | Yes |
| 63 | SPC24 |  |  | 91 | 0.544 | 0.3303 | Yes |
| 64 | CENPM |  |  | 92 | 0.544 | 0.3351 | Yes |
| 65 | NCAPH |  |  | 94 | 0.543 | 0.3379 | Yes |
| 66 | PBK |  |  | 95 | 0.540 | 0.3427 | Yes |
| 67 | CSRP2 |  |  | 96 | 0.539 | 0.3475 | Yes |
| 68 | DEPDC1 |  |  | 97 | 0.536 | 0.3522 | Yes |
| 69 | XRCC2 |  |  | 98 | 0.530 | 0.3569 | Yes |
| 70 | SKA3 |  |  | 99 | 0.525 | 0.3615 | Yes |
| 71 | TMEM106C |  |  | 100 | 0.523 | 0.3661 | Yes |
| 72 | RRM1 |  |  | 103 | 0.520 | 0.3668 | Yes |
| 73 | MXD3 |  |  | 104 | 0.519 | 0.3714 | Yes |
| 74 | CMC2 |  |  | 105 | 0.516 | 0.3760 | Yes |
| 75 | CDKN3 |  |  | 108 | 0.513 | 0.3766 | Yes |
| 76 | CEP135 |  |  | 109 | 0.513 | 0.3811 | Yes |
| 77 | UBE2T |  |  | 110 | 0.512 | 0.3856 | Yes |
| 78 | CDCA3 |  |  | 111 | 0.511 | 0.3901 | Yes |
| 79 | DIAPH3 |  |  | 112 | 0.510 | 0.3946 | Yes |
| 80 | TUBB4B |  |  | 114 | 0.507 | 0.3972 | Yes |
| 81 | RAD21 |  |  | 115 | 0.506 | 0.4016 | Yes |
| 82 | BIRC5 |  |  | 116 | 0.504 | 0.4061 | Yes |
| 83 | CLSPN |  |  | 117 | 0.503 | 0.4105 | Yes |
| 84 | DTYMK |  |  | 118 | 0.503 | 0.4150 | Yes |
| 85 | CEP152 |  |  | 119 | 0.502 | 0.4194 | Yes |
| 86 | ASF1B |  |  | 120 | 0.501 | 0.4238 | Yes |
| 87 | PSMC3IP |  |  | 122 | 0.495 | 0.4263 | Yes |
| 88 | DUT |  |  | 124 | 0.494 | 0.4287 | Yes |
| 89 | E2F8 |  |  | 125 | 0.493 | 0.4330 | Yes |
| 90 | TMPO |  |  | 127 | 0.492 | 0.4354 | Yes |
| 91 | ITGB3BP |  |  | 128 | 0.490 | 0.4397 | Yes |
| 92 | LMNB2 |  |  | 129 | 0.488 | 0.4440 | Yes |
| 93 | SKA1 |  |  | 131 | 0.487 | 0.4464 | Yes |
| 94 | SHCBP1 |  |  | 133 | 0.484 | 0.4487 | Yes |
| 95 | GMNN |  |  | 135 | 0.484 | 0.4510 | Yes |
| 96 | C21orf58 |  |  | 142 | 0.470 | 0.4434 | Yes |
| 97 | CDCA4 |  |  | 148 | 0.461 | 0.4377 | Yes |
| 98 | FANCD2 |  |  | 149 | 0.461 | 0.4418 | Yes |
| 99 | ORC6 |  |  | 150 | 0.461 | 0.4459 | Yes |
| 100 | KIF22 |  |  | 152 | 0.459 | 0.4480 | Yes |
| 101 | NCAPD3 |  |  | 153 | 0.458 | 0.4520 | Yes |
| 102 | CDC45 |  |  | 154 | 0.456 | 0.4560 | Yes |
| 103 | MCM8 |  |  | 158 | 0.454 | 0.4542 | Yes |
| 104 | CENPQ |  |  | 163 | 0.446 | 0.4503 | Yes |
| 105 | RPA1 |  |  | 165 | 0.445 | 0.4523 | Yes |
| 106 | TK1 |  |  | 167 | 0.444 | 0.4542 | Yes |
| 107 | BRIP1 |  |  | 168 | 0.443 | 0.4582 | Yes |
| 108 | PIMREG |  |  | 169 | 0.443 | 0.4621 | Yes |
| 109 | ATAD5 |  |  | 170 | 0.442 | 0.4660 | Yes |
| 110 | BRCA1 |  |  | 171 | 0.440 | 0.4699 | Yes |
| 111 | BRCA2 |  |  | 174 | 0.436 | 0.4698 | Yes |
| 112 | RBBP8 |  |  | 176 | 0.436 | 0.4717 | Yes |
| 113 | DEPDC1B |  |  | 177 | 0.436 | 0.4756 | Yes |
| 114 | MKI67 |  |  | 179 | 0.436 | 0.4774 | Yes |
| 115 | FEN1 |  |  | 180 | 0.435 | 0.4813 | Yes |
| 116 | USP1 |  |  | 185 | 0.430 | 0.4773 | Yes |
| 117 | MIS18BP1 |  |  | 186 | 0.430 | 0.4811 | Yes |
| 118 | MGME1 |  |  | 188 | 0.428 | 0.4829 | Yes |
| 119 | BARD1 |  |  | 189 | 0.427 | 0.4867 | Yes |
| 120 | MND1 |  |  | 193 | 0.424 | 0.4845 | Yes |
| 121 | MIS18A |  |  | 196 | 0.421 | 0.4843 | Yes |
| 122 | CCDC15 |  |  | 197 | 0.419 | 0.4880 | Yes |
| 123 | MCM4 |  |  | 199 | 0.418 | 0.4898 | Yes |
| 124 | DSN1 |  |  | 202 | 0.413 | 0.4895 | Yes |
| 125 | CCP110 |  |  | 204 | 0.411 | 0.4912 | Yes |
| 126 | PEG10 |  |  | 206 | 0.410 | 0.4928 | Yes |
| 127 | TCF19 |  |  | 207 | 0.410 | 0.4965 | Yes |
| 128 | ZWINT |  |  | 212 | 0.407 | 0.4922 | Yes |
| 129 | LIG1 |  |  | 214 | 0.407 | 0.4939 | Yes |
| 130 | CCNE2 |  |  | 215 | 0.406 | 0.4975 | Yes |
| 131 | H2AFZ |  |  | 216 | 0.405 | 0.5010 | Yes |
| 132 | HSPB11 |  |  | 218 | 0.401 | 0.5026 | Yes |
| 133 | MNS1 |  |  | 219 | 0.401 | 0.5062 | Yes |
| 134 | FANCA |  |  | 227 | 0.395 | 0.4960 | Yes |
| 135 | TIMELESS |  |  | 228 | 0.395 | 0.4994 | Yes |
| 136 | PKMYT1 |  |  | 233 | 0.390 | 0.4951 | Yes |
| 137 | POC1A |  |  | 234 | 0.390 | 0.4985 | Yes |
| 138 | EZH2 |  |  | 236 | 0.388 | 0.5000 | Yes |
| 139 | VRK1 |  |  | 238 | 0.388 | 0.5015 | Yes |
| 140 | CDC6 |  |  | 239 | 0.388 | 0.5049 | Yes |
| 141 | PARPBP |  |  | 240 | 0.387 | 0.5083 | Yes |
| 142 | TRIP13 |  |  | 241 | 0.387 | 0.5117 | Yes |
| 143 | PCNA |  |  | 242 | 0.386 | 0.5151 | Yes |
| 144 | RTKN2 |  |  | 248 | 0.379 | 0.5087 | Yes |
| 145 | POLD3 |  |  | 252 | 0.377 | 0.5061 | Yes |
| 146 | RAD18 |  |  | 256 | 0.373 | 0.5036 | Yes |
| 147 | RFC5 |  |  | 260 | 0.370 | 0.5010 | Yes |
| 148 | CENPJ |  |  | 261 | 0.370 | 0.5042 | Yes |
| 149 | TUBB |  |  | 263 | 0.368 | 0.5055 | Yes |
| 150 | GINS4 |  |  | 265 | 0.367 | 0.5068 | Yes |
| 151 | ASRGL1 |  |  | 266 | 0.366 | 0.5100 | Yes |
| 152 | CENPW |  |  | 267 | 0.366 | 0.5133 | Yes |
| 153 | TUBG1 |  |  | 268 | 0.365 | 0.5165 | Yes |
| 154 | TUBA1C |  |  | 270 | 0.362 | 0.5177 | Yes |
| 155 | SMC1A |  |  | 274 | 0.361 | 0.5151 | Yes |
| 156 | CHAF1A |  |  | 276 | 0.359 | 0.5163 | Yes |
| 157 | MMS22L |  |  | 277 | 0.359 | 0.5195 | Yes |
| 158 | ANP32E |  |  | 280 | 0.358 | 0.5187 | Yes |
| 159 | GINS1 |  |  | 283 | 0.357 | 0.5180 | Yes |
| 160 | PSIP1 |  |  | 284 | 0.357 | 0.5211 | Yes |
| 161 | ZWILCH |  |  | 292 | 0.353 | 0.5105 | No |
| 162 | HAUS8 |  |  | 293 | 0.353 | 0.5136 | No |
| 163 | RFC4 |  |  | 295 | 0.351 | 0.5148 | No |
| 164 | SUZ12 |  |  | 297 | 0.349 | 0.5159 | No |
| 165 | DTL |  |  | 305 | 0.345 | 0.5053 | No |
| 166 | PRKDC |  |  | 306 | 0.344 | 0.5083 | No |
| 167 | CENPN |  |  | 309 | 0.343 | 0.5074 | No |
| 168 | ABHD3 |  |  | 310 | 0.343 | 0.5104 | No |
| 169 | FAM111B |  |  | 314 | 0.342 | 0.5076 | No |
| 170 | OIP5 |  |  | 315 | 0.341 | 0.5106 | No |
| 171 | DEK |  |  | 316 | 0.341 | 0.5136 | No |
| 172 | PHF19 |  |  | 319 | 0.339 | 0.5127 | No |
| 173 | CSE1L |  |  | 321 | 0.338 | 0.5137 | No |
| 174 | RFC3 |  |  | 322 | 0.338 | 0.5167 | No |
| 175 | OLFM1 |  |  | 325 | 0.337 | 0.5158 | No |
| 176 | RFC2 |  |  | 331 | 0.333 | 0.5089 | No |
| 177 | EMP2 |  |  | 333 | 0.333 | 0.5099 | No |
| 178 | HIRIP3 |  |  | 336 | 0.331 | 0.5089 | No |
| 179 | BTG3 |  |  | 337 | 0.331 | 0.5118 | No |
| 180 | UBR7 |  |  | 340 | 0.330 | 0.5108 | No |
| 181 | NFATC2IP |  |  | 343 | 0.328 | 0.5098 | No |
| 182 | PHGDH |  |  | 344 | 0.328 | 0.5127 | No |
| 183 | NRGN |  |  | 348 | 0.326 | 0.5097 | No |
| 184 | LRRCC1 |  |  | 349 | 0.326 | 0.5126 | No |
| 185 | HAT1 |  |  | 352 | 0.324 | 0.5116 | No |
| 186 | TMEM237 |  |  | 353 | 0.324 | 0.5144 | No |
| 187 | LMNB1 |  |  | 363 | 0.321 | 0.4997 | No |
| 188 | WDR34 |  |  | 366 | 0.321 | 0.4986 | No |
| 189 | RBBP7 |  |  | 367 | 0.321 | 0.5014 | No |
| 190 | PARP2 |  |  | 372 | 0.319 | 0.4964 | No |
| 191 | NCAPH2 |  |  | 377 | 0.315 | 0.4913 | No |
| 192 | PIN1 |  |  | 379 | 0.314 | 0.4922 | No |
| 193 | POLA2 |  |  | 380 | 0.314 | 0.4949 | No |
| 194 | DCK |  |  | 383 | 0.313 | 0.4938 | No |
| 195 | TCEAL9 |  |  | 385 | 0.312 | 0.4946 | No |
| 196 | PRIM2 |  |  | 391 | 0.311 | 0.4876 | No |
| 197 | HAUS2 |  |  | 393 | 0.311 | 0.4883 | No |
| 198 | TEDC1 |  |  | 399 | 0.309 | 0.4813 | No |
| 199 | LRR1 |  |  | 410 | 0.304 | 0.4644 | No |
| 200 | PRADC1 |  |  | 421 | 0.299 | 0.4475 | No |
| 201 | DSCC1 |  |  | 422 | 0.299 | 0.4501 | No |
| 202 | GAMT |  |  | 434 | 0.295 | 0.4312 | No |
| 203 | RECQL4 |  |  | 443 | 0.294 | 0.4181 | No |
| 204 | TFDP1 |  |  | 444 | 0.294 | 0.4207 | No |
| 205 | TEX30 |  |  | 448 | 0.294 | 0.4175 | No |
| 206 | CBX5 |  |  | 457 | 0.291 | 0.4044 | No |
| 207 | TMUB1 |  |  | 462 | 0.289 | 0.3991 | No |
| 208 | SAE1 |  |  | 463 | 0.289 | 0.4017 | No |
| 209 | YEATS4 |  |  | 476 | 0.286 | 0.3807 | No |
| 210 | CHEK1 |  |  | 477 | 0.286 | 0.3832 | No |
| 211 | CDT1 |  |  | 486 | 0.284 | 0.3701 | No |
| 212 | CHTF18 |  |  | 493 | 0.282 | 0.3608 | No |
| 213 | CENPH |  |  | 501 | 0.281 | 0.3496 | No |
| 214 | PSMC3 |  |  | 506 | 0.280 | 0.3443 | No |
| 215 | IKBIP |  |  | 508 | 0.279 | 0.3448 | No |
| 216 | WDHD1 |  |  | 513 | 0.278 | 0.3394 | No |
| 217 | LSM3 |  |  | 522 | 0.277 | 0.3262 | No |
| 218 | DNMT1 |  |  | 523 | 0.277 | 0.3286 | No |
| 219 | RAB23 |  |  | 524 | 0.277 | 0.3311 | No |
| 220 | SLF1 |  |  | 526 | 0.276 | 0.3316 | No |
| 221 | SAC3D1 |  |  | 529 | 0.276 | 0.3301 | No |
| 222 | NFIC |  |  | 531 | 0.276 | 0.3306 | No |
| 223 | ALYREF |  |  | 546 | 0.273 | 0.3056 | No |
| 224 | ACYP1 |  |  | 551 | 0.273 | 0.3002 | No |
| 225 | CALU |  |  | 552 | 0.272 | 0.3026 | No |
| 226 | DONSON |  |  | 559 | 0.271 | 0.2932 | No |
| 227 | NUP85 |  |  | 563 | 0.270 | 0.2897 | No |
| 228 | GGH |  |  | 564 | 0.270 | 0.2921 | No |
| 229 | CDK2 |  |  | 565 | 0.270 | 0.2945 | No |
| 230 | CTNNAL1 |  |  | 570 | 0.269 | 0.2891 | No |
| 231 | RPA3 |  |  | 584 | 0.267 | 0.2660 | No |
| 232 | SLC43A3 |  |  | 590 | 0.265 | 0.2585 | No |
| 233 | IDH2 |  |  | 598 | 0.263 | 0.2472 | No |
| 234 | KPNB1 |  |  | 600 | 0.263 | 0.2475 | No |
| 235 | CEP57 |  |  | 605 | 0.262 | 0.2420 | No |
| 236 | RAD1 |  |  | 607 | 0.262 | 0.2424 | No |
| 237 | AKR7A2 |  |  | 610 | 0.262 | 0.2408 | No |
| 238 | KDELC2 |  |  | 612 | 0.261 | 0.2411 | No |
| 239 | UBA2 |  |  | 626 | 0.259 | 0.2180 | No |
| 240 | CHCHD3 |  |  | 628 | 0.259 | 0.2183 | No |
| 241 | ARL6IP6 |  |  | 629 | 0.258 | 0.2206 | No |
| 242 | SCCPDH |  |  | 638 | 0.257 | 0.2072 | No |
| 243 | EXOSC8 |  |  | 647 | 0.256 | 0.1938 | No |
| 244 | UHRF1 |  |  | 652 | 0.255 | 0.1882 | No |
| 245 | PIGX |  |  | 657 | 0.255 | 0.1827 | No |
| 246 | RDX |  |  | 658 | 0.255 | 0.1849 | No |
| 247 | PXMP2 |  |  | 663 | 0.253 | 0.1793 | No |
| 248 | MYBL1 |  |  | 678 | 0.251 | 0.1541 | No |
| 249 | METRN |  |  | 695 | -0.273 | 0.1252 | No |
Table: GSEA details [plain text format]

  

Fig 2: DOROSZKO\_U3180MG\_CLUSTER\_3: Random ES distribution      
 Gene set null distribution of ES for **DOROSZKO\_U3180MG\_CLUSTER\_3**
